# Examining the Use of an Artificial Intelligence Model to Diagnose Influenza: Development and Validation Study

**DOI:** 10.2196/38751

**Published:** 2022-12-23

**Authors:** Sho Okiyama, Memori Fukuda, Masashi Sode, Wataru Takahashi, Masahiro Ikeda, Hiroaki Kato, Yusuke Tsugawa, Masao Iwagami

**Affiliations:** 1 Aillis, Inc Tokyo Japan; 2 Japanese Red Cross Medical Center Tokyo Japan; 3 Department of Health Informatics Graduate School of Medicine, School of Public Health Kyoto University Kyoto Japan; 4 Department of Palliative Therapy Cancer Institute Hospital of Japanese Foundation for Cancer Research Tokyo Japan; 5 Tokyo Medical and Dental University Tokyo Japan; 6 Division of General Internal Medicine and Health Services Research David Geffen School of Medicine at The University of California, Los Angeles Los Angeles, CA United States; 7 Department of Health Services Research University of Tsukuba Tsukuba Japan; 8 Faculty of Epidemiology and Population Health London School of Hygiene and Tropical Medicine London United Kingdom

**Keywords:** influenza, physical examination, pharynx, deep learning, diagnostic prediction

## Abstract

**Background:**

The global burden of influenza is substantial. It is a major disease that causes annual epidemics and occasionally, pandemics. Given that influenza primarily infects the upper respiratory system, it may be possible to diagnose influenza infection by applying deep learning to pharyngeal images.

**Objective:**

We aimed to develop a deep learning model to diagnose influenza infection using pharyngeal images and clinical information.

**Methods:**

We recruited patients who visited clinics and hospitals because of influenza-like symptoms. In the training stage, we developed a diagnostic prediction artificial intelligence (AI) model based on deep learning to predict polymerase chain reaction (PCR)–confirmed influenza from pharyngeal images and clinical information. In the validation stage, we assessed the diagnostic performance of the AI model. In additional analysis, we compared the diagnostic performance of the AI model with that of 3 physicians and interpreted the AI model using importance heat maps.

**Results:**

We enrolled a total of 7831 patients at 64 hospitals between November 1, 2019, and January 21, 2020, in the training stage and 659 patients (including 196 patients with PCR-confirmed influenza) at 11 hospitals between January 25, 2020, and March 13, 2020, in the validation stage. The area under the receiver operating characteristic curve for the AI model was 0.90 (95% CI 0.87-0.93), and its sensitivity and specificity were 76% (70%-82%) and 88% (85%-91%), respectively, outperforming 3 physicians. In the importance heat maps, the AI model often focused on follicles on the posterior pharyngeal wall.

**Conclusions:**

We developed the first AI model that can accurately diagnose influenza from pharyngeal images, which has the potential to help physicians to make a timely diagnosis.

## Introduction

### Background

According to the Global Burden of Disease Study 2016, the global burden of influenza is substantial. In the study, the disease was estimated to cause 39.1 million acute lower respiratory infection episodes and 58,200 deaths [1]. It has been estimated that influenza is responsible for 291,243 to 645,832 seasonal respiratory deaths (4.0-8.8 per 100,000 individuals) annually [2]. Timely and accurate diagnosis of influenza has the potential to prevent widespread transmission of the virus within the population, and subsequent epidemics and pandemics, in addition to the unnecessary prescription of antibiotics in primary care, which is a cause of emerging antibiotic-resistant bacteria. Moreover, early intervention, such as hydration and antiviral drugs, is expected to reduce the mortality risk among high-risk patients, including the older adults and individuals with comorbidities.

The COVID-19 pandemic and surge in the use of telemedicine highlighted the importance of accurately diagnosing influenza infection without increasing the risk of spreading the virus through physical interaction. The gold-standard method for diagnosing influenza infection is the reverse transcription–polymerase chain reaction (RT-PCR) of nasopharyngeal aspirates or swabs [3,4]; however, RT-PCR is not easily performed in primary care, and the result turnaround time could delay timely diagnosis and preventive or treatment interventions. A more commonly used test is the rapid immunochromatographic antigen detection test; however, its validity is modest compared with RT-PCR and varies across studies [5,6]. Neither of these tests can be performed through telemedicine, and the sensitivity and specificity of diagnosing influenza using clinical information only are suboptimal [7,8]. Given the recent increase in the number of patients being diagnosed through telemedicine, an alternative influenza test that can be conducted through telemedicine is warranted.

### Objectives

To address this important knowledge gap, we developed a deep learning model to diagnose influenza infection using pharyngeal images and clinical information. We tested the performance of the artificial intelligence (AI) model for diagnostic prediction using data from the real-world patient population and compared it with the diagnostic performance of 3 physicians. We also investigated the regions of the pharynx on which the AI model focused to differentiate between individuals with and without influenza infection.

## Methods

### Pilot Study to Develop a Medical Camera to Capture Standardized Pharyngeal Images

**Figure 1:**
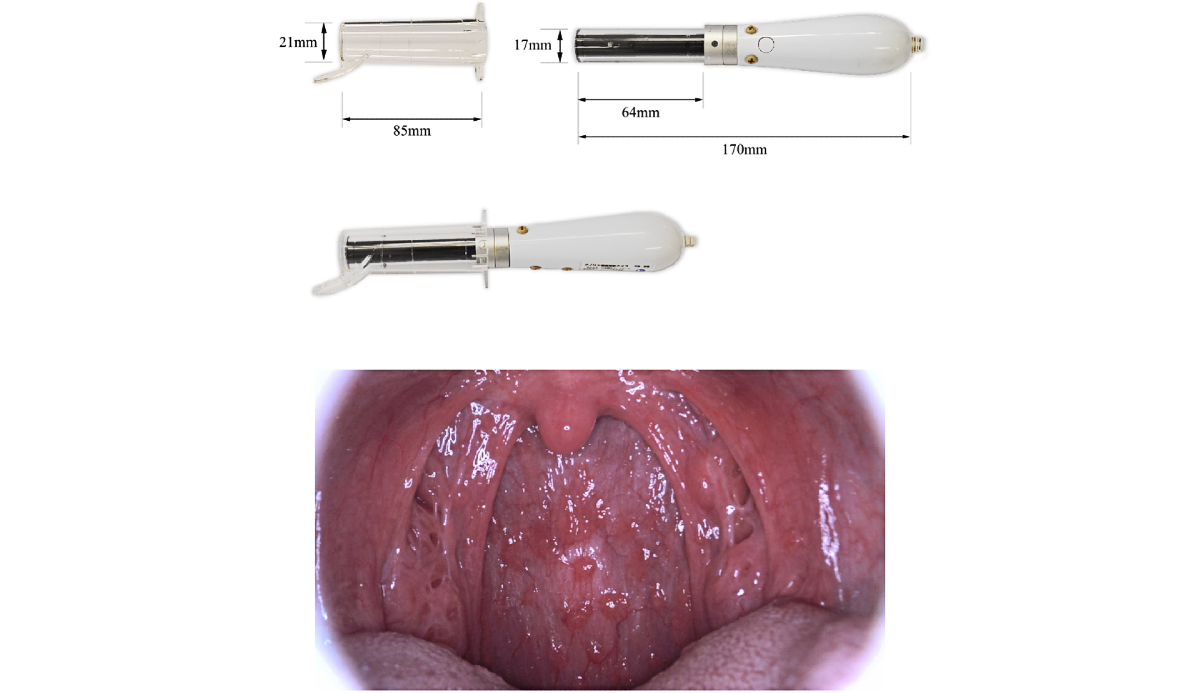
Presentation of the artificial intelligence–assisted camera and a representative pharyngeal image of a patient with polymerase chain reaction–confirmed influenza infection.

For our pilot study, we recruited 4765 patients aged 6 to 90 years with influenza-like symptoms, and they visited 37 clinics or hospitals between November 28, 2018, and February 4, 2019 (registered as jRCTs032180041). To capture images of the pharynx in a standardized manner, we developed a pharyngeal camera with a light-emitting diode light source and a disposable clear camera cover to hold down the tongue of patients (Figure 1). In this pilot study, we adjusted the size of the pharyngeal camera and tongue depressors to make them suitable for many patients. The device contained a full high-definition digital camera and was connected via Wi-Fi to a cloud service for the analysis of pharyngeal images, together with clinical information. During this pilot study, we improved the image quality of the camera in terms of resolution, brightness, and contrast. Specifically, we reduced the view angle appropriately to reduce distortion and improve the resolution because the angle was excessive. We also placed an imaging sensor near the tip of the camera to avoid light attenuation and ensure image brightness. In addition, we improved the image contrast by coating the clear camera cover with antifogging material to prevent fogging caused by exhalation. We used a rapid continuous shooting function to obtain high-quality pharyngeal images in a short time while avoiding motion blur. The camera could capture an image every 0.3 seconds, and 30 sequential images were captured per shot.

### Study Design and Participants

This study included a training stage (registered as jRCTs032190120) and a validation stage (registered as Pharmaceuticals and Medical Devices Agency clinical trial identification code AI-02-01). We enrolled patients with influenza-like symptoms who visited clinics or hospitals and satisfied the following inclusion and exclusion criteria at 64 hospitals between November 1, 2019, and January 21, 2020, in the training stage, and 11 hospitals between January 25, 2020, and March 13, 2020, in the validation stage. A list of study sites is provided in Table S1 in Multimedia Appendix 1.

The inclusion criteria were as follows: (1) patients who provided written consent by themselves or their parents (if they were aged <18 years) to participate in the study, (2) those aged ≥6 years, and (3) those who satisfied at least one of the following 4 conditions in the training stage and at least 2 in the validation stage: first, body temperature of ≥37.0 °C; second, systematic influenza-like symptoms, such as joint pain, muscle pain, headache, tiredness, and appetite loss; third, respiratory symptoms, such as cough, sore throat, and nasal discharge or congestion; and fourth, an episode of close contact with patients with influenza or influenza-like symptoms within 3 days, or in any other scenario in which the consulting physician suspected influenza infection. The exclusion criteria included the following: (1) patients with fluctuating teeth; (2) those with severe oral lesions; (3) those with severe nausea; (4) those with difficulty in opening the mouth sufficiently for the use of the camera (eg, small mouth, temporomandibular joint pain, incompatibility of dentures, disturbed consciousness, or respiratory failure); (5) those who had participated in another clinical trial within 7 days before this study, those who were scheduled to participate in another clinical trial (excluding postmarketing surveillance), or those with difficulty in follow-up owing to mental, family, social, geographic, or other reasons; (6) pediatric patients who clearly did not agree to participate in the study; and (7) those judged to be inappropriate to participate in the study by the responsible physician at each site. In addition, we excluded patients with only poor-quality images from the analysis.

In the training stage, we aimed to collect clinical information and pharyngeal images from patients with RT-PCR–confirmed influenza-positive and influenza-negative results in a ratio of approximately 1:1 to enable the most efficient supervised learning of the AI model. There is no consensus on the size of the samples (ie, number of patients) that should be used to train an AI model; thus, we arbitrarily set the size to 7500 patients, including 3750 patients with RT-PCR–positive results and 3750 patients with RT-PCR–negative results. In the validation stage, we aimed to determine the lower bound of the 95% one-sided CI of sensitivity to achieve ≥70% and that of specificity to achieve ≥85%. With a 1-sided *P* value of 5% and power of 85%, assuming an actual sensitivity of 80% and specificity of 90% as suggested by our training stage, we calculated the required sample sizes to be 137 for patients with RT-PCR–positive results and 323 for RT-PCR–negative results. Therefore, we planned to stop the recruitment of study participants on the day when 150 patients with positive results and 350 patients with negative results were obtained.

In Japan, the first case of SARS-CoV-2 infection (COVID-19) was reported on January 15, 2020, and the first wave of the pandemic occurred in late March 2020. During the study period, in the validation stage, we asked the participating clinics and hospitals to report any suspected cases of COVID-19 in the study participants. There were no such reports from any study site throughout the study, which suggests that our study sample was not affected by the COVID-19 pandemic.

### Collection of Pharyngeal Images, Clinical Information, and Nasopharyngeal Specimens

In addition to the pharyngeal images of the study participants, the following clinical information was obtained using a standardized case report form based on electronic data capture: age; sex; time (hours) from symptom onset; highest body temperature before study site visit; episode of close contact; status and date of the most recent influenza vaccination; use of antipyretics; subjective symptoms, including tiredness, appetite loss, chill, sweating, joint pain, muscle pain, headache, nasal discharge or congestion, cough, sore throat, and digestive symptoms; and objective findings by the consulting physicians at study sites, including body temperature, pulse rate, and tonsillar findings (tonsillitis, white moss, and redness).

Furthermore, nasopharyngeal swabbing was conducted to obtain nasopharyngeal specimens from the participants, which were sent to the central clinical laboratory (LSI Medience Corporation) for RT-PCR testing, which is the gold standard (reference standard) for the diagnosis of influenza infection. We standardized the process of collecting the nasopharyngeal specimens among the study sites using our own Japanese manual (not publicly available).

### Development of the AI Model to Predict RT-PCR–Confirmed Influenza

We developed an ensemble AI model (version FLU2021.06) to predict the probability of RT-PCR–confirmed influenza using pharyngeal images and clinical information (Figure S1 in Multimedia Appendix 1). This model consists of 3 main machine learning models: a multiview convolutional neural network (MV-CNN), a multimodal convolutional neural network (MM-CNN), and boosting models. In the training stage, we trained these 3 types of machine learning models and integrated them using ridge regression [9] into the ensemble AI model.

First, we trained the MV-CNN using SE-ResNext-50 as an image feature extractor, which was pretrained on ImageNet [10,11]. The MV-CNN architecture used several pharyngeal images that contained views from various angles [12]. On pharyngeal imaging, the tongue and uvula often overlap with the posterior pharyngeal wall. The MV-CNN addressed this issue by gathering information from various image angles. From 30 (or more if several shots were taken) sequential images, 1 to 5 of the most appropriate images per patient were selected as the input to the MV-CNN using an automatic image quality evaluation system. We determined the number of input images by considering the MV-CNN performance and the memory size limitation of the graphics processor units. Although, in general, the MV-CNN performs better with more input images, the memory size of the graphics processor unit constrains the number of images. If the number of selected images was <5, we padded them with uninformative images filled with zeros, similar to zero padding in the boundary region of an image. To quantify the visual image quality criteria, we trained the image quality evaluation system that used a lightweight CNN model [13] in the training stage using human-annotated visual image quality criteria (eg, visibility of the posterior pharyngeal wall, brightness, focus, motion blur, and exhalation fog) defined by one of the authors (MF) who is a physician. The input images for the MV-CNN were resized and then augmented (eg, flipped, rotated, blurred, and contrast-changed) to improve the accuracy and generalization performance. To prevent overfitting, we used well-established training strategies, including batch normalization, learning rate decay, and cross-validation. To manage various pharyngeal magnification rates, we trained the MV-CNNs with multiple image sizes and combined their scores by averaging them.

Second, we developed the MM-CNN based on the MV-CNN to process both multiview pharyngeal images and clinical information as input data [14,15]. In detail, we extended the final classification layer of the MV-CNN and connected it to the neural network to manage clinical information. The image feature extractor of the MM-CNN was initialized using trained MV-CNN weights. Then, we applied the same training and ensemble strategies as those used for the MV-CNN.

Third, we trained boosting models based on the prediction results of MV-CNN and clinical information. We selected LightGBM and CatBoost as the boosting models [16,17]. Finally, the probability of influenza was obtained by integrating each prediction from the MV-CNN, MM-CNN, and boosting models using ridge regression. We trained the ridge regression weights using cross-validation. The probability of influenza was obtained by averaging all the folds of the ridge regression model predictions.

### Statistical Analysis

In the training stage, we compared the clinical characteristics of the study participants according to the RT-PCR test results (positive or negative) using *t* tests (2 tailed) for continuous variables with a normal distribution (age, highest body temperature before the study site visit, body temperature at visit, and pulse rate), Mann-Whitney *U* test for continuous variables with a nonnormal distribution (time from symptom onset), and chi-square tests for categorical variables. We repeated these analyses in the validation stage.

In the training stage, using a 5-fold cross-validation method, we conducted a receiver operating characteristic (ROC) curve analysis to measure the discrimination ability of (1) the probability score of the MV-CNN, which uses only pharyngeal images in the prediction; (2) the probability score of the clinical information AI, which is an AI model that uses all the aforementioned clinical information, except for the pharyngeal images, in the prediction; and (3) the probability score of the ensemble AI model using both the pharyngeal images and clinical information. We also measured the reclassification ability of the pharyngeal images by comparing the clinical information AI model and the ensemble AI model by calculating the continuous reclassification improvement and integrated discrimination improvement [18].

In the validation stage, we also conducted ROC analysis and calculated the sensitivity, specificity, positive predictive value (PPV), and negative predictive value (NPV) for influenza infection, according to a selected cutoff point.

We performed statistical analysis using R software (version 4.1.1; R Foundation) and Python software (version 3.8.5; Python Software Foundation). *P* values of <.05 were considered statistically significant. A third-party organization (Statcom Co Ltd, Tokyo, Japan) performed the sample size estimation and calculation of the area under the ROC curve (AUROC) and validity (sensitivity, specificity, PPV, and NPV) in the validation stage. To avoid the post hoc adjustment of the developed AI model to fit the validation data in the regulatory approval process, the authors were prohibited from directly touching the validation data or conducting additional analyses in the validation stage. Therefore, any other analyses (eg, the calculation of AUROC for pharyngeal images and clinical information independently or for the MV-CNN, MM-CNN, and boosting model separately) were not possible in the validation stage.

### Additional Analysis

We conducted 4 types of additional analyses. First, we compared the performance of the AI-assisted diagnostic camera with that of the 3 physicians. For this analysis, we used the existing data (pharyngeal images and clinical information) of 200 patients (100 patients with RT-PCR–positive results and 100 with RT-PCR–negative results), which were randomly selected from study participants in the training stage. A total of 3 physicians among the authors (SO, MF, and M Ikeda), who were blinded to the patients’ identifiers and their RT-PCR test results, assessed the data to assign an influenza prediction score between 0 and 1 (ie, between 0% and 100%). As there is generally no established practice or criteria for physicians to diagnose influenza from pharyngeal images and clinical information, the 3 physicians were asked to guess the probability of influenza infection for each patient, as they usually do in their actual clinical practice. We applied the diagnostic prediction AI model to the existing data and compared the AUROC of the diagnostic prediction AI model with that of each physician and the average prediction score of the 3 physicians. We recalculated the AUROC of the AI model for the 200 patients for a fair comparison.

Second, we attempted to interpret the mechanisms of the MV-CNN prediction to differentiate between influenza cases and noninfluenza cases using pharyngeal images. We modified the guided gradient-weighted class activation mapping for the MV-CNN to visualize the importance heat maps. The aim was to determine the focus area of MV-CNN when differentiating between patients with RT-PCR–positive and RT-PCR–negative results. We used the same data set of 200 patients (100 patients with RT-PCR–positive and 100 with RT-PCR–negative results) that we used in the first additional analysis. To quantify and interpret the importance heat maps, 2 physicians among the authors (MF and M Ikeda) independently determined whether the MV-CNN highlighted each part of the pharynx (classified into 5 parts: lateral pharyngeal bands, posterior pharyngeal wall, palatal arch, tonsils, and follicles) for each patient. When the 2 physicians made different judgments (ie, presence vs absence of highlighting by the MV-CNN), a consensus was reached through discussion between them. Consequently, for each part of the pharynx, we calculated the proportion of patients with images highlighted by the MV-CNN among the 100 patients with RT-PCR–positive results and 100 RT-PCR–negative results and compared the groups using chi-square tests.

Third, as a post hoc experiment, using the 200 samples, we compared the performance of our final model (ie, the ridge regression ensemble model) with the performance of each of the component models: the MV-CNN, MM-CNN, and boosting models.

Finally, as another post hoc experiment, we compared the performance of the MV-CNN model with the proposed backbone (SE-ResNext-50) and that of various CNN backbones, that is, ResNet-50, ResNeXt-50 (32×4d), EfficientNet-B0, and DenseNet-121, which were available at the time of our model development.

### Ethics Approval

The ethics committee of Hattori Clinic approved the pilot study and the training study, and the validation study was approved by the ethics committee of Takahashi Clinic, Kobori Central Clinical, and Haradoi Hospital.

## Results

### Training Stage

Figure S2 in Multimedia Appendix 1 shows the flowchart of patient selection during the training stage. We obtained informed consent from 9029 patients with influenza-like symptoms who visited one of 64 clinics or hospitals between November 1, 2019, and January 21, 2020. Among them, 199 patients (2.20%) experienced nausea during the examination when pharyngeal images were being captured, including 1 (0.01%) patient with severe nausea and 14 (0.16%) patients who vomited. We did not complete the image-capturing procedure for these 15 patients (0.17%). Among the remaining 9014 patients, we selected 7831 patients (mean age 33.8, SD 18.4 years; female patients: 3901/7831, 50%) with 25,168 high-quality images (out of approximately 300,000 images), which consisted of 3733 (47.67%) patients with influenza RT-PCR–positive results with 12,154 (48.29%) pharyngeal images and 4098 (52.33%) patients with RT-PCR–negative results with 13,014 (51.71%) pharyngeal images. Table 1 compares the clinical characteristics of the patients based on the RT-PCR test results. Compared with the patients with RT-PCR–negative results, the patients with RT-PCR–positive results yielded the following: the average age was slightly lower; the time from symptom onset to the study site visit was shorter; the proportion of close contact, use of antipyretics, and most subjective symptoms were higher; and the temperature and pulse rate were higher, whereas the proportion of recent influenza vaccinations, digestive symptoms, and tonsillar findings were lower. There was no difference in the proportions of sex and sore throat between the groups.

Using the training data set, we established the ensemble AI model to estimate the probability of influenza in individual patients. The feature importance of each variable in the LightGBM and CatBoost models is shown in Figures S3 and S4 in Multimedia Appendix 1, which suggest that pharyngeal images were the most important variable in the diagnostic prediction AI model, followed by body temperature and cough.

**Figure 2:**
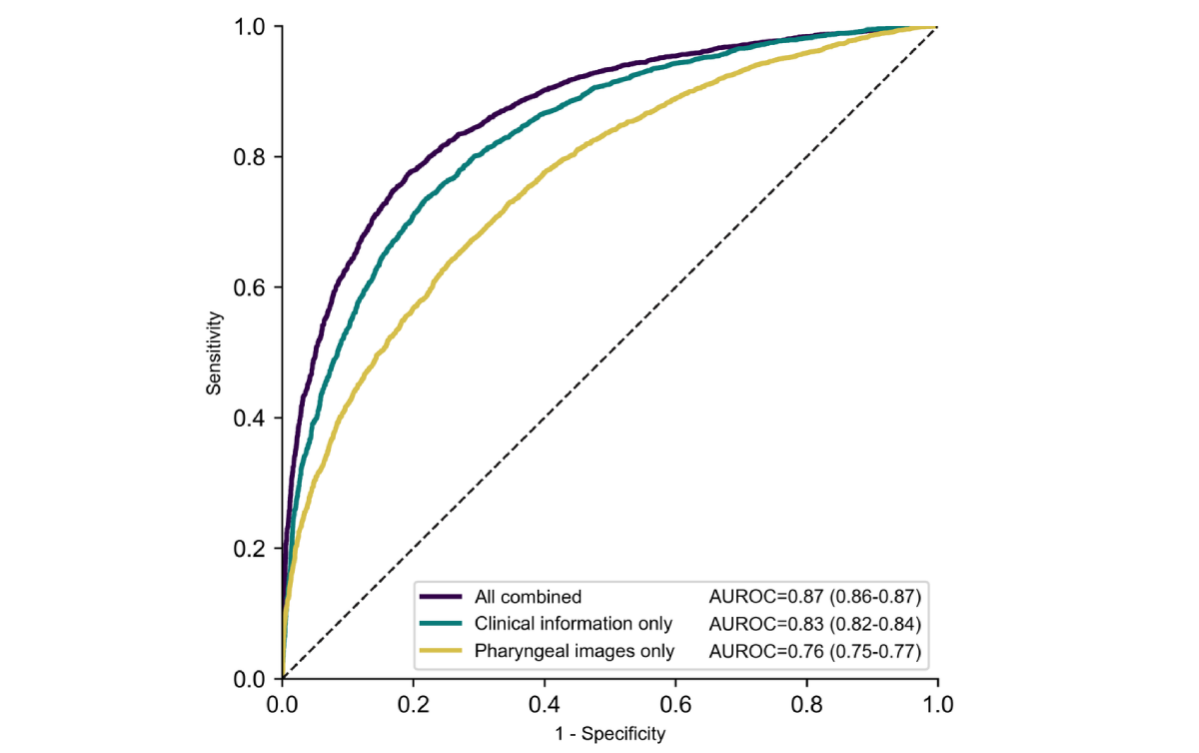
Receiver operating characteristic curves of the diagnostic prediction models in the 5-fold cross-validation of the training data set. In the figure, all combined indicates ensemble artificial intelligence (AI) model using pharyngeal images and clinical information; pharyngeal images only indicates multiview convolutional neural network using multiple pharyngeal images; clinical information only indicates ensemble AI model without pharyngeal image information. AUROC: area under the receiver operating characteristic.

In the 5-fold cross-validation, the AUROC of the MV-CNN probability score for pharyngeal images was 0.76 (95% CI 0.75-0.77) and that of the AI model with clinical information (ie, all the clinical information in Table 1) was 0.83 (95% CI 0.82-0.84; Figure 2). The AUROC of the diagnostic prediction AI model with pharyngeal images and clinical information was 0.87 (95% CI 0.86-0.87), which means that the AUROC significantly increased because of the addition of pharyngeal images to the AI model with clinical information (*P*<.001). Regarding reclassification ability, the continuous reclassification improvement was 0.25 (95% CI 0.22-0.29) among patients with RT-PCR–positive results and 0.33 (95% CI 0.30-0.36) among patients with RT-PCR–negative results and the integrated discrimination improvement was 0.08 (95% CI 0.07-0.08), which also indicate that the accuracy of the diagnostic prediction AI model significantly improved because of the addition of pharyngeal images to the AI model with clinical information.

**Table 1:** Characteristics of the study participants with or without reverse transcription–polymerase chain reaction (RT-PCR)–confirmed influenza.

Characteristics		Participants in the training stage							Participants in the validation stage					
		All (n=7831)	RT-PCR test result					All (n=659)			RT-PCR test result			
			Positive (n=3733)	Negative (n=4098)	*P* value					Positive (n=196)		Negative (n=463)	*P* value	
Age (years), mean (SD)		33.8 (18.4)	33.0 (18.5)	34.5 (18.4)	<.001		33.3 (17.6)			30.4 (18.6)		34.5 (17.0)	.008	
**Sex** **, n (%)**						.54								.54
	Male	3930 (50.2)	1887 (50.5)	2043 (49.9)			318 (48.3)			91 (46.4)		227 (49.0)		
	Female	3901 (49.8)	1846 (49.5)	2055 (50.1)			341 (51.7)			105 (53.6)		236 (51.0)		
Time from onset (hours), mean (SD)		31.2 (25.3)	28.3 (20.6)	33.8 (28.6)	<.001		27.5 (31.2)			24.6 (10.8)		28.7 (36.5)	.67	
Highest BT^a^ before visit (°C), mean (SD)		38.2 (0.9)	38.6 (0.8)	38.0 (0.9)	<.001		38.2 (0.8)			38.6 (0.7)		38.0 (0.8)	<.001	
Close contact, n (%)		2520 (32.2)	1687 (45.2)	833 (20.3)	<.001		208 (31.6)			120 (61.2)		88 (19.0)	<.001	
Recent influenza vaccination, n (%)		2873 (36.7)	1248 (33.4)	1625 (39.7)	<.001		278 (42.2)			73 (37.2)		205 (44.3)	.09	
Use of antipyretics, n (%)		2975 (38)	1530 (41)	1445 (35.3)	<.001		297 (45.1)			95 (48.5)		202 (43.6)	.25	
**Subjective symptoms** **, n (%)**														
	Tiredness	5937 (75.8)	3010 (80.6)	2927 (71.4)	<.001		506 (76.8)			159 (81.1)		347 (74.9)	.09	
	Appetite loss	3361 (42.9)	1823 (48.8)	1538 (37.5)	<.001		259 (39.3)			96 (49)		163 (35.2)	<.001	
	Chill	4215 (53.8)	2231 (59.8)	1984 (48.4)	<.001		338 (51.3)			115 (58.7)		223 (48.2)	.01	
	Sweating	2188 (27.9)	1128 (30.2)	1060 (25.9)	<.001		206 (31.3)			60 (30.6)		146 (31.5)	.82	
	Joint pain	3735 (47.7)	1992 (53.4)	1743 (42.5)	<.001		316 (48)			103 (52.6)		213 (46)	.12	
	Muscle pain	2362 (30.2)	1276 (34.2)	1086 (26.5)	<.001		192 (29.1)			62 (31.6)		130 (28.1)	.36	
	Headache	4725 (60.3)	2414 (64.7)	2311 (56.4)	<.001		403 (61.2)			126 (64.3)		277 (59.8)	.28	
	Nasal discharge or congestion	4472 (57.1)	2202 (59)	2270 (55.4)	.001		410 (62.2)			134 (68.4)		276 (59.6)	.03	
	Cough	5219 (66.6)	3053 (81.8)	2166 (52.9)	<.001		384 (58.3)			161 (82.1)		223 (48.2)	<.001	
	Sore throat	4928 (62.9)	2353 (63)	2575 (62.8)	.86		440 (66.8)			126 (64.3)		314 (67.8)	.38	
	Digestive symptoms	1298 (16.6)	558 (14.9)	740 (18.1)	<.001		127 (19.3)			30 (15.3)		97 (21)	.09	
**Objective findings**														
	BT at visit (°C), mean (SD)	37.6 (0.9)	38.0 (0.9)	37.3 (0.8)	<.001		37.5 (0.9)			37.9 (0.9)		37.3 (0.8)	<.001	
	Pulse rate, mean (SD)	95.0 (17.8)	100.2 (17.7)	90.3 (16.6)	<.001		93.8 (17.7)			100.8 (18.6)		90.9 (16.4)	<.001	
	Tonsillitis, n (%)	1238 (15.8)	529 (14.2)	709 (17.3)	<.001		63 (9.6)			8 (4.1)		55 (11.9)	.002	
	Tonsillar white moss, n (%)	126 (1.6)	17 (0.5)	109 (2.7)	<.001		23 (3.5)			1 (0.5)		22 (4.8)	.007	
	Tonsillar redness, n (%)	1292 (16.5)	540 (14.5)	752 (18.4)	<.001		69 (10.5)			13 (6.6)		56 (12.1)	.04	

^a^BT: body temperature.

### Validation Stage

Figure S5 in Multimedia Appendix 1 shows the flowchart of patient selection during the validation stage. In the validation stage, we obtained informed consent from 706 patients with influenza-like symptoms who visited one of 11 clinics or hospitals between January 25, 2020, and March 13, 2020, which comprised a safety analysis set. Of the 706 patients, 12 (1.7%) felt nauseous during the examination when the pharyngeal images were being captured, including 1 patient (0.1%) with severe nausea for whom we did not complete the image-taking procedure. In addition, 33 patients (4.7%) did not satisfy the predefined criteria of the protocol for the full analysis set, mostly because of the difficulties in saving pharyngeal images at the study sites. Furthermore, 13 (1.8%) patients were excluded from the automated image quality evaluation system that removed low-quality pharyngeal images. Thus, we used the pharyngeal images and clinical information of the remaining 659 patients (mean age 33.3 years, SD 17.6 years; female patients: 341/659, 51.7%) for the validation stage analysis. Similar to the training stage, compared with noncases, the RT-PCR–confirmed cases yielded the following results: the average age was slightly lower; the proportion of close contact and several subjective symptoms (tiredness, chills, nasal discharge or obstruction, and cough) was higher; and the temperature (both before the clinic or hospital visit and on site) and pulse rate were higher, whereas the proportion of tonsillar findings was lower (Table 1).

**Figure 3:**
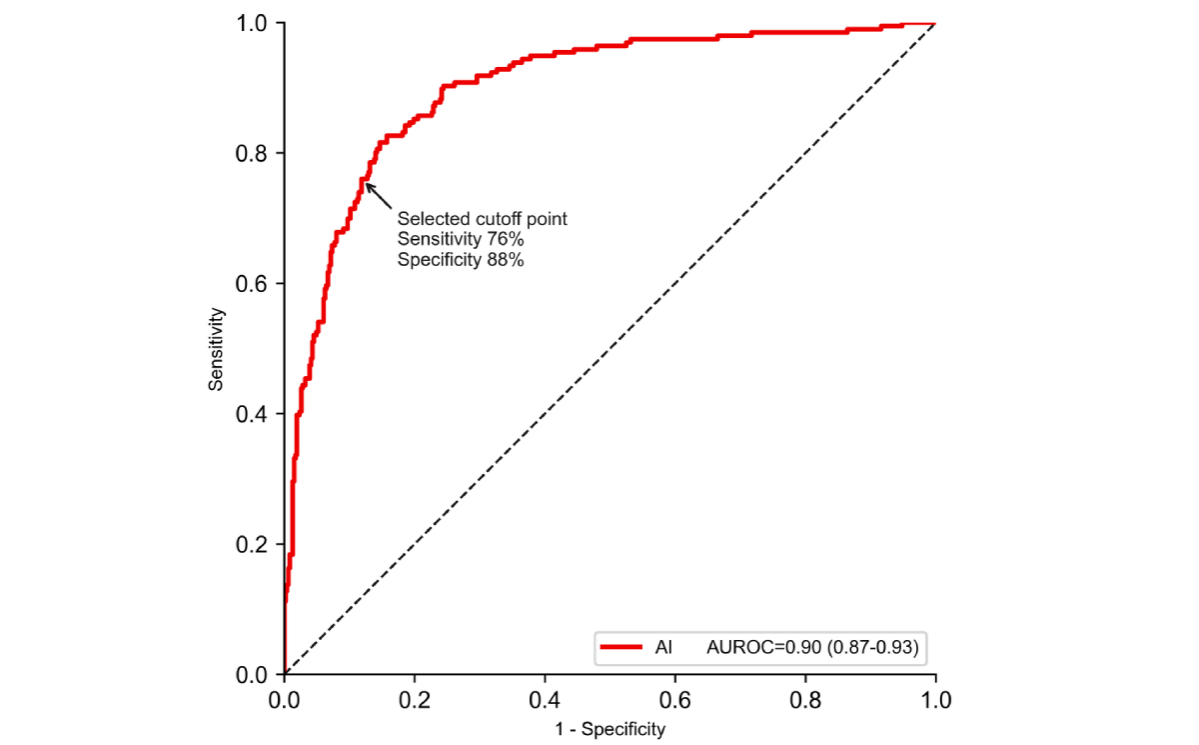
Receiver operating characteristic curve for the diagnostic prediction model in the validation data set. AI: artificial intelligence; AUROC: area under the receiver operating characteristic curve.

In the validation stage, the AUROC of the diagnostic prediction AI model was 0.90 (95% CI 0.87-0.93). At a selected cutoff point on the ROC curve (Figure 3), the sensitivity and specificity were 76% (95% CI 70%-82%) and 88% (95% CI 85%-91%), respectively, and the PPV and NPV were 73% (95% CI 69%-79%) and 90% (95% CI 87%-92%), respectively (Table 2).

**Table 2:** Validity of the artificial intelligence (AI)–assisted device compared with the gold-standard diagnosis of influenza virus infection based on reverse transcription–polymerase chain reaction (RT-PCR).

		Influenza virus infection based on RT-PCR			Total, n		Values, % (95% CI)	
		True positive	True negative			PPV^a^, % (95% CI)		NPV^b^, % (95% CI)
**Prediction by the AI-assisted device^c^, n**								
	Positive	149	55	204		73 (67-79)		N/A^d^
	Negative	47	408	455		N/A		90 (87-92)
Total, n		196	463	659		N/A		N/A
Sensitivity, % (95% CI)		76 (70-82)	N/A	N/A		N/A		N/A
Specificity, % (95% CI)		N/A	88 (85-91)	N/A		N/A		N/A

^a^PPV: positive predictive value.

^b^NPV: negative predictive value.

^c^According to the selected cutoff point on the receiver operating characteristic curve of the diagnostic prediction model of the AI-assisted device shown in Figure 3.

^d^N/A: not applicable.

### Additional Analysis

**Figure 4:**
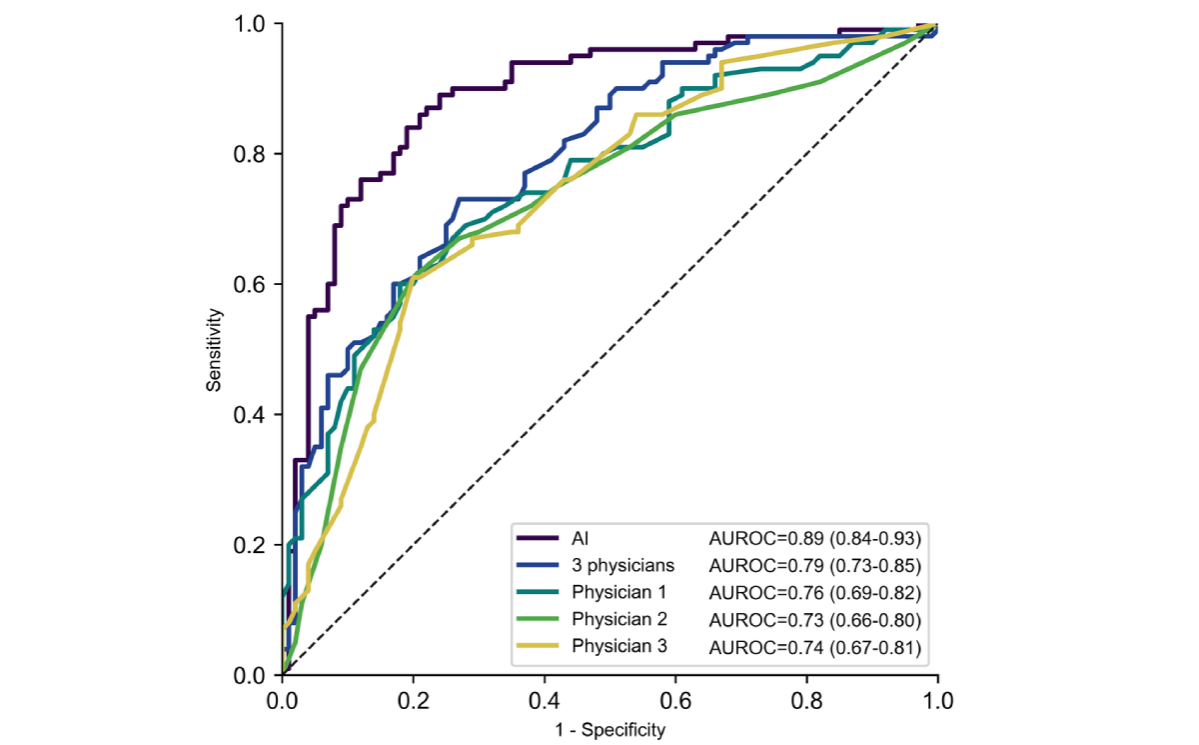
Receiver operating characteristic curves for the diagnostic prediction artificial intelligence model and 3 physicians. In the figure, AI indicates ensemble AI model using pharyngeal images and clinical information. The AI model was the same as that used in the validation stage. However, the AUROC was slightly different because of the small sample size used in the additional analysis. AI: artificial intelligence; AUROC: area under the receiver operating characteristic curve. 3 physicians: average prediction score of the 3 physicians.

In our additional analysis, among the 200 randomly selected patients (100 patients with RT-PCR–positive results and 100 with RT-PCR–negative results), the AUROC of the diagnostic prediction AI model was 0.89 (95% CI 0.84-0.93), which was higher than that of each of the 3 physicians (0.76, 0.73, and 0.74). It was also higher than that of the average prediction score of the 3 physicians (0.79, 95% CI 0.73-0.85; Figure 4).

**Figure 5:**
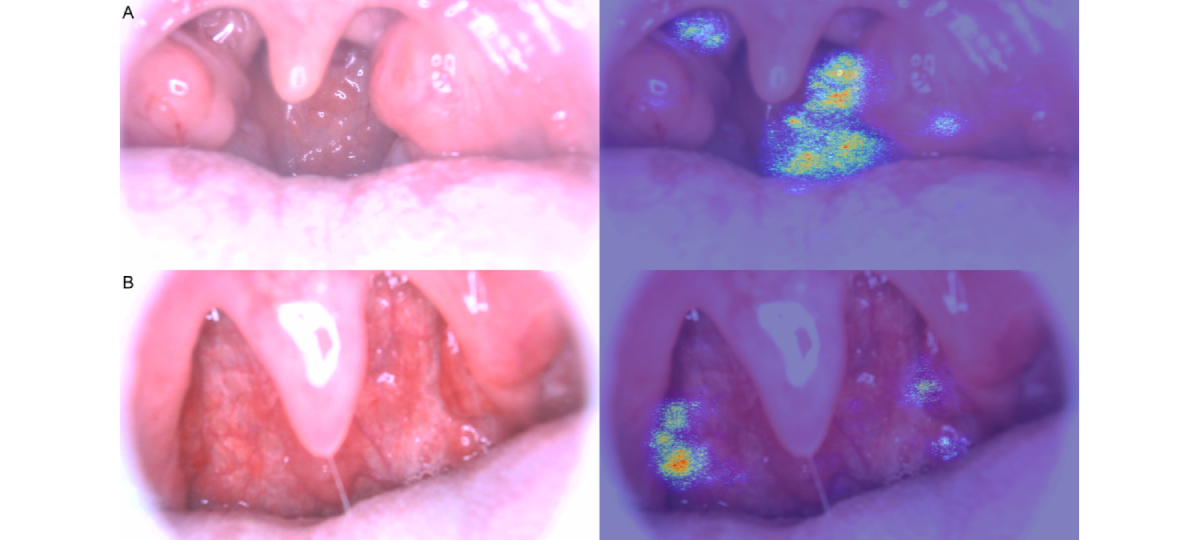
Examples of pharyngeal images (left) and those highlighted using the importance heatmaps (right). These importance heat maps show areas in which the artificial intelligence (AI) model focused on differentiating between reverse transcriptase–polymerase chain reaction (RT-PCR)–positive cases and RT-PCR–negative cases. In example A, the AI model focused on follicles. In example B, the AI model focused on the lateral pharyngeal bands.

Figure S6 in Multimedia Appendix 1 and Figure 5 show examples of the pharyngeal images and those highlighted using the importance heat maps. An assessment of the importance heat maps for the 200 patients (100 patients with RT-PCR–positive results and 100 with RT-PCR–negative results) conducted by 2 physicians showed that the proportion of patients with AI model–highlighted images of follicles on the posterior pharyngeal wall was significantly different between the patients with RT-PCR–positive and RT-PCR–negative results (73% vs 38%; *P*<.001), which suggests that the AI model often focused on these parts (Figure S7 in Multimedia Appendix 1).

Finally, our post hoc experiments suggested that the performance of our final model (ie, the ridge regression ensemble model) was superior or similar to (at least not inferior to) the performance of each component model (Table S2 in Multimedia Appendix 1). In addition, the backbone model proposed in our final model was superior to various CNN backbones (Table S3 in Multimedia Appendix 1).

## Discussion

### Principal Findings

In this study, we developed an AI-assisted diagnostic camera using a diagnostic prediction model for influenza (Multimedia Appendix 2). In the training stage, we found that the pharyngeal images contributed significantly to the improvement of the diagnostic prediction AI model compared with the clinical information AI model. In the validation stage, the AUROC of the diagnostic prediction AI model was 0.90 (95% CI 0.87-0.93), with a sensitivity and specificity of 76% (95% CI 70%-82%) and 88% (95% CI 85%-91%), respectively. In our additional analysis, the AI-assisted camera performed better than the 3 physicians in predicting influenza. Furthermore, in the importance heat maps, we found that the AI model often focused on follicles to differentiate between patients with RT-PCR–positive and RT-PCR–negative results.

Clinical characteristics associated with RT-PCR–confirmed influenza infection among people with influenza-like symptoms were examined in 2 previous studies [7,8]. Both studies concluded that fever and cough were the best predictors of influenza diagnosis. However, the sensitivity and specificity of the combination of these 2 factors were suboptimal, at 78% and 55% in one study [7] and 64% and 67% in another study, respectively [8]. In our study, considering the feature importance of each variable in the LightGBM and CatBoost models (Figures S3 and S4 in Multimedia Appendix 1), body temperature and cough were highly ranked among clinical information, whereas the feature importance of pharyngeal images was even larger than the highly ranked clinical information.

Recently, several AI-assisted diagnostic prediction models have been proposed for influenza diagnosis [19-22]. In a single-center study from Japan, researchers reported a machine learning–based infection screening system that incorporates a random tree algorithm that uses vital signs [19]. The researchers reported a sensitivity of 81% to 96% and NPV of 81% to 96% in their training data sets (they did not report specificity and PPV); however, they did not validate the performance of the model outside the center. Researchers at the University of Pittsburgh Medical Center Health System reported machine learning classifiers for influenza detection from free-text reports of the emergency department [20,21]. Among the 31,268 emergency department reports from 4 hospitals, the AUROCs of the 7 machine learning classifiers for influenza detection ranged from 0.88 to 0.93 [21], which was better than an expert-built Bayesian model [20]. These studies were also limited because performance outside the health care system of the University of Pittsburgh was unknown. More recently, a Korean study reported an influenza screening system based on deep learning using a combination of epidemiological and patient-generated health data from a mobile health app [22]. However, the gold standard in the study was the clinical diagnosis of influenza at a clinic reported by app users instead of laboratory-confirmed influenza. Notably, none of the previous studies included an assessment of pharyngeal images in their diagnostic prediction models [19-22]. The novelty of our study is that we have developed the first AI-assisted diagnostic camera for influenza and prospectively validated its performance through a Good Clinical Practice-based clinical trial process.

We showed that pharyngeal images significantly improved the discrimination and reclassification abilities of the diagnostic prediction AI model. In addition, we considered the mechanisms by which the AI model differentiated between true influenza cases and noninfluenza cases using pharyngeal images. To the best of our knowledge, there is no established approach to quantitatively scale the regions of images on which the AI model focuses. Indeed, most previous studies on AI-assisted diagnostic cameras showed only representative images highlighted using gradient-weighted class activation mapping or saliency maps to speculate on the possible mechanisms of AI classification [23-25]. In our study, we attempted to quantify these regions by calculating the proportion of patients with images highlighted by the AI model for each part of the pharynx among the patients with RT-PCR–positive and RT-PCR–negative results. Consequently, we found that the AI model mainly focused on follicles on the posterior pharyngeal wall. Notably, this finding is in line with previous case reports and case series that suggest that the follicles on the posterior pharyngeal wall are specific to influenza infection and are useful for the diagnosis of influenza [26-29]. Physical examination, including visual inspection of the pharynx, generally requires the experience of individual physicians, and physical examination skills may vary widely among physicians. Our study suggests that AI could minimize the variation and may help to standardize physical examination skills among physicians. In addition, when attempting to discriminate between diseases, doctors may be able to learn where to focus on their visual examination using AI systems.

### Limitations

Our study has some limitations. First, we recruited participants with influenza-like symptoms from a large number of clinics and hospitals in Japan to increase the generalizability of our study. However, there may be a country or cultural difference in terms of people with influenza-like symptoms seeking medical care from health care providers. In Japan, with its universal health care coverage, people have relatively easy and timely access to clinics and hospitals compared with those in other countries. Therefore, generalizing our findings to different clinical care settings in different countries requires caution and independent assessment. Second, our additional analysis of the comparison between the AI-assisted diagnostic camera and the 3 physicians was not planned in the study protocols (jRCTs032190120 and Pharmaceuticals and Medical Devices Agency clinical trial identification code AI-02-01); however, these physicians were blinded to the patients’ identifiers and their RT-PCR results. Finally, in addition to pharyngeal images, we collected as many relevant clinical variables (suggested in previous large studies [7,8]) as possible to establish an accurate diagnostic prediction AI model. However, there may be other useful variables for the prediction of true influenza diagnosis that we did not collect in our study. For example, in some studies, researchers have suggested that the population-level trend of influenza outbreaks in an area is useful for predicting an individual patient’s influenza infection [22]. Further improvement of the AI-assisted diagnostic camera by including additional variables, as well as an improvement of the AI models to analyze pharyngeal images, is justified.

### Conclusions

In conclusion, we developed the first AI-assisted diagnostic camera for influenza and prospectively validated its high performance. We found that the AI model often focused on follicles, which confirmed previous case reports and series suggesting that visual inspection of the pharynx would help in the diagnosis of influenza infection.

## Appendix

Multimedia Appendix 1 Supplementary Figures 1-7 and Tables 1-3.

Multimedia Appendix 2 Concept video of pharyngeal examination and artificial intelligence (AI) diagnosis using our pharyngeal AI camera system “nodoca”.

## Abbreviations

AI: artificial intelligence
AUROC: area under the ROC curve
MM-CNN: multimodal convolutional neural network
MV-CNN: multiview convolutional neural network
NPV: negative predictive value
PPV: positive predictive value
ROC: receiver operating characteristic
RT-PCR: reverse transcription–polymerase chain reaction
