# Supplementary material for: Examining the Use of an Artificial Intelligence Model to Diagnose Influenza: Development and Validation Study
Source: J Med Internet Res. 2022 Dec 23;24(12):e38751. doi: 10.2196/38751 (PMC9823578; doi:10.2196/38751)
Supplement: Multimedia Appendix 1 [file jmir_v24i12e38751_app1.docx]

**Supplementary Table 1. List of study sites**

| Pilot stage (37 sites): Adachi Kyosai Hospital; Ahiko Otolaryngology Clinic; Asahiyama Hospital; Clinic Kashiwanoha; Dozenkai Clinic; Ebisu Clinic; Fukazawa Clinic; Fukuda Clinic (Internal medicine); Funai Ear Nose Throat Clinic; Iguchi Clinic; Irie Child Clinic; Kamei Internal Medicine and Respiratory Clinic; Kamoike ENT Allergy Clinic; Kanagawa Himawari Clinic; Kaneko Clinic; Kanna Hospital; Kawaguchi Kogyo General Hospital; Megumi Clinic; Minami Clinic; Miyazaki RC Clinic; Moriyama Otolaryngology Clinic; Musashino General Hospital; Nishimura Clinic; Nomura Clinic; Okura Otolaryngology Clinic; Rokujizo General Hospital; Ryuto Otolaryngology Clinic; Sawayama Clinic; Shinnakama Hospital; Someya Clinic; Takahashi Clinic; Terada Clinic, Respiratory Medicine and General Practice; Yamagata Clinic; Yamashita Child Clinic; Yasuda Clinic; Yokoyama Children’s Clinic; Yoshimura Child Clinic |
| --- |
| Training stage (64 sites): Aozora Children’s Hospital; Association of Healthcare Corporation Meiko-kai Ohishi Naika Clinic; Clinic Kashiwanoha; Den-en-tyofu Family Clinic; Dozenkai Clinic; Ebisu Clinic; Eifukuchoekimae Minnano Clinic; Fukuda Clinic (Internal medicine); Himeno Hospital; Ikeda Naika Clinic; Ito ENT Clinic; Kamoike ENT Allergy Clinic; Kanagawa Himawari Clinic; Kikumori Ear, Nose and Throat Clinic; Kimura Clinic; Kumeda Clinic; Kunisaki Makoto Clinic; Maekawa Medical Clinic; Marunouchi Hospital; Mashiki Clinic; Matsuda Pediatric Clinic; Medical Corporation Association Kanwakai Musashikoganei Clinic; Medical Corporation Hitomikai Motomachi Takatsuka Naika Clinic; Medical Corporation Houmankai Umezu Clinic; Medical Corporation Segawa Hospital; Medical Corporation Yuhokai Miho-Clinic; Medical Square Kuhonji Clinic; Megumi Clinic; Miuraiin; Miyanosawa Clinic of Internal Medicine and Cardiology; Miyazaki RC Clinic; Morimoto ENT Clinic; Moriyama Otolaryngology Clinic; Nakamura Cardiovascular Clinic; Nakano Clinic; Nanko Clinic; Nishiyamadou Keiwa Hospital; Nomura Clinic; Okura Otolaryngology Clinic; Primula Clinic; Saino Clinic; Sakata ENT Clinic; Sakura Hospital; Sannou Yamate Clinic; Sasaki Clinic; Sato ENT Clinic; Shimada Clinic; Shirao Clinic of Pediatrics and Pediatric Allergy; Someya Clinic; Sone Clinic Shinjuku; Suzuki Clinic; Suzuki Internal Medicine Clinic; Tanabe Pediatrics; Terada Clinic, Respiratory medicine and General Practice; Ueyama Child Clinic; Umemotokodomo Clinic; Uranishi Clinic; Wada Clinic; Yaesu Clinic; Yamada Clinic; Yamaichi Building Medical Clinic; Yokoyama Children’s Clinic; Yoshimura Child Clinic; YOSHIMURA CLiNiC |
| Validation stage (11 sites): Dozenkai Clinic; Fukuda Clinic (Internal medicine); Himeno Hospital; Kimura Clinic; Nakano Clinic; Sato ENT Clinic; Shimada Clinic; Terada Clinic, Respiratory Medicine and General Practice; Ueyama Child Clinic; Uranishi Clinic; Yaesu Clinic |

**Supplementary Figure 1. Structure of the ensemble AI model used to predict the probability of RT-PCR-confirmed influenza using pharyngeal images and clinical information**


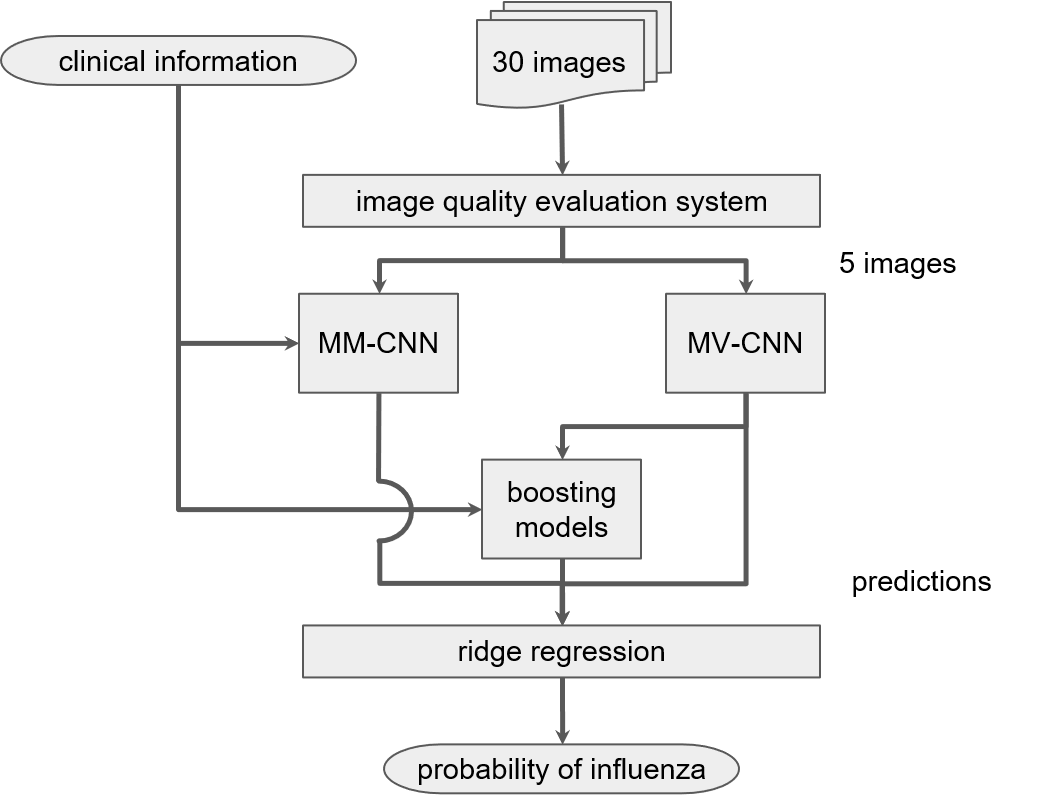


**Supplementary Figure 2. Flow chart for patient selection in the training stage**

**
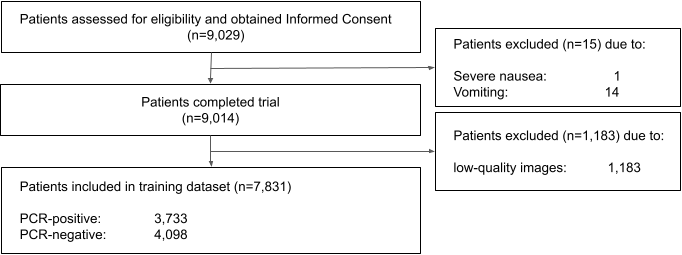
**

Note: Image quality was assessed using the automated image quality evaluation system.

**Supplementary Figure 3. Feature importance of pharyngeal images and clinical information for the LightGBM model**


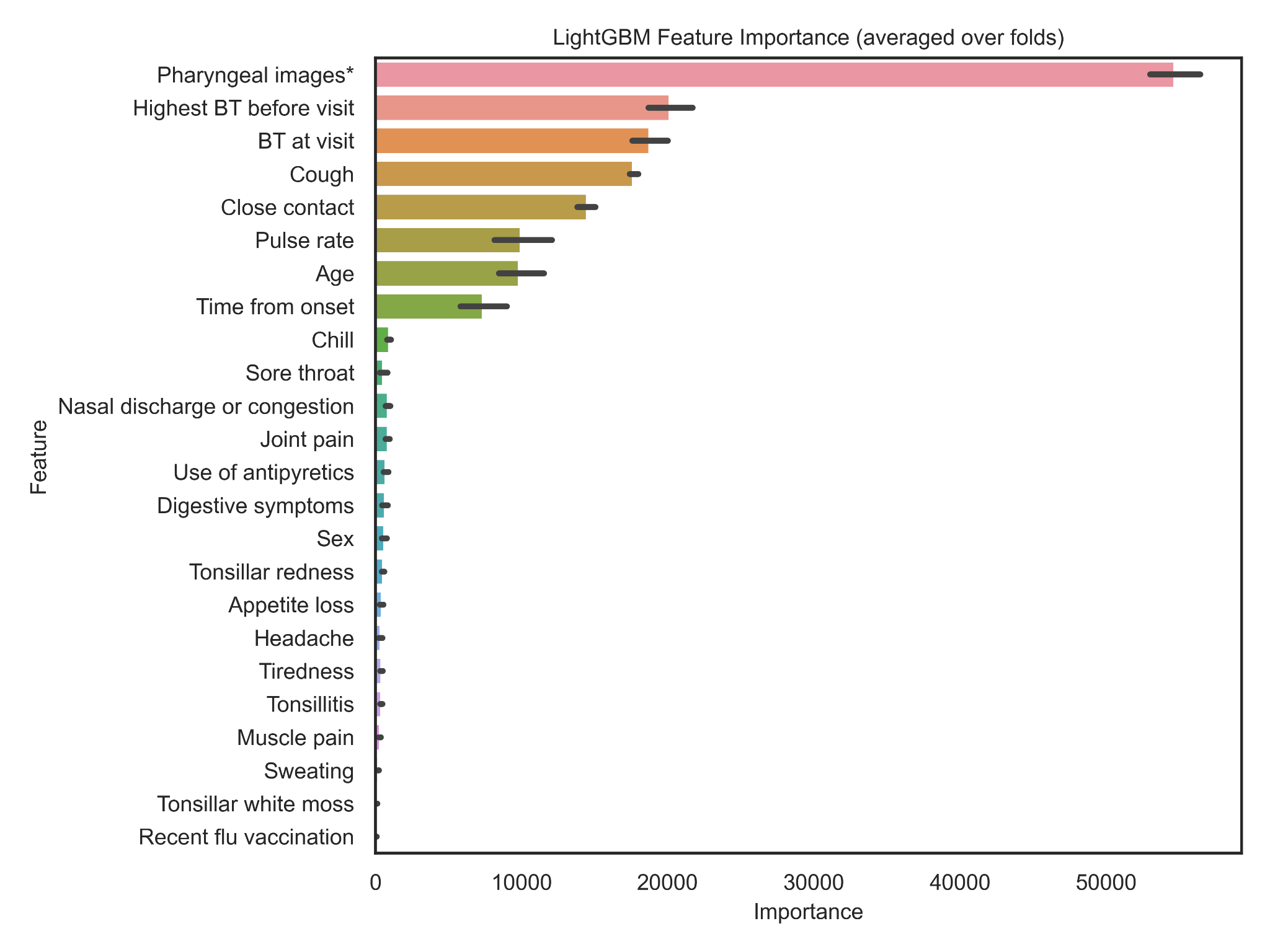


Abbreviation: BT: body temperature

*multi-view convolutional neural network (MV-CNN) influenza probability based on pharyngeal images

**Supplementary Figure 4. Feature importance of pharyngeal images and clinical information for the CatBoost model**

**
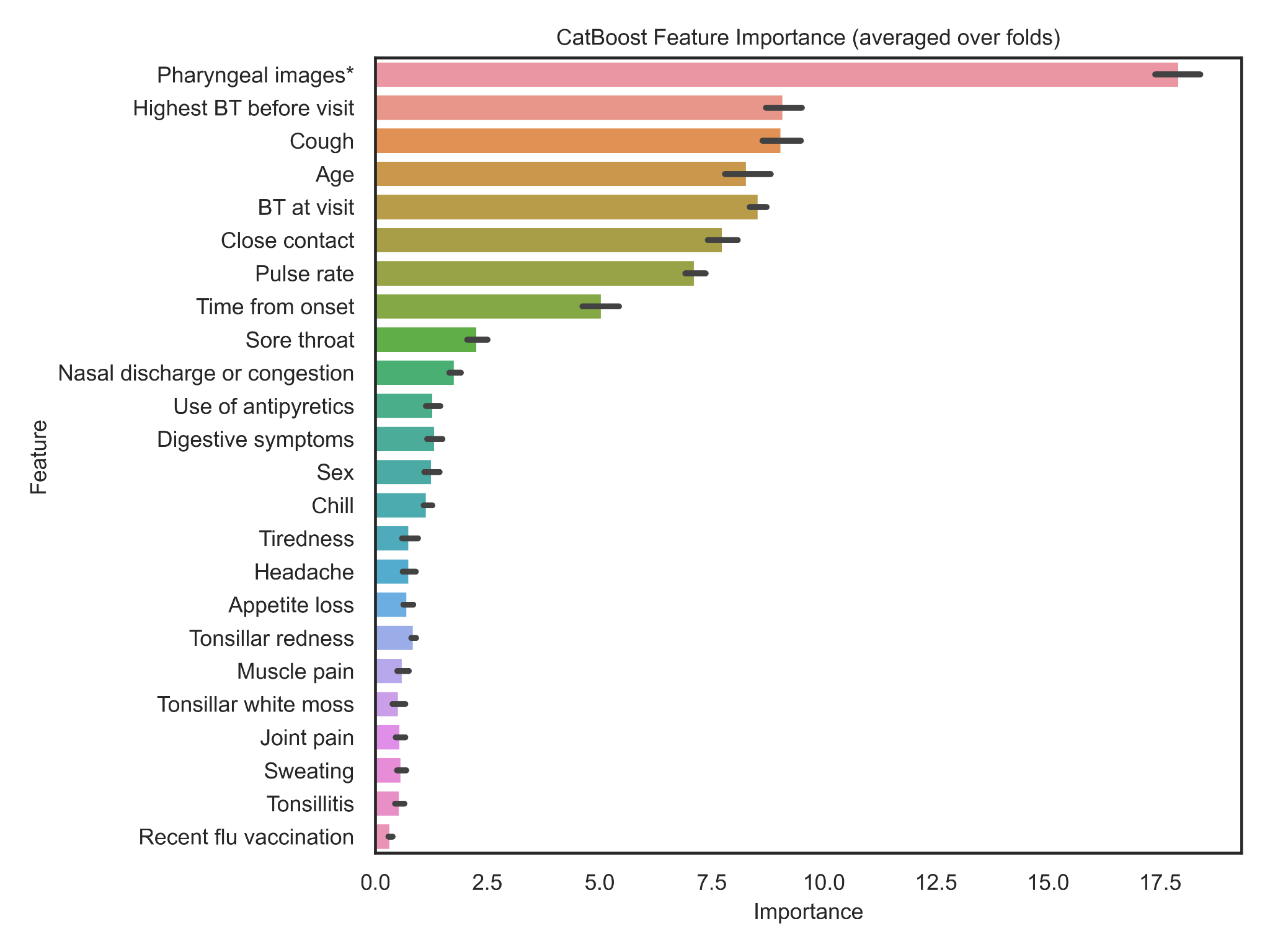
**Abbreviation: BT: body temperature

*multi-view convolutional neural network (MV-CNN) influenza probability based on pharyngeal images

**Supplementary Figure 5. Flow chart for patient selection in the validation stage**

**
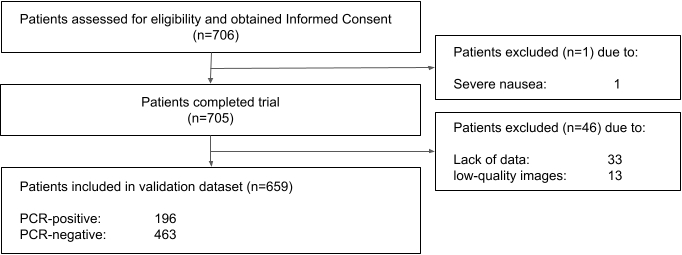
**

Note: Image quality was assessed using the automated image quality evaluation system.

**Supplementary Figure 6. Other examples of pharyngeal images and those highlighted using the importance heatmaps**

Positive case:

Posterior pharyngeal wall


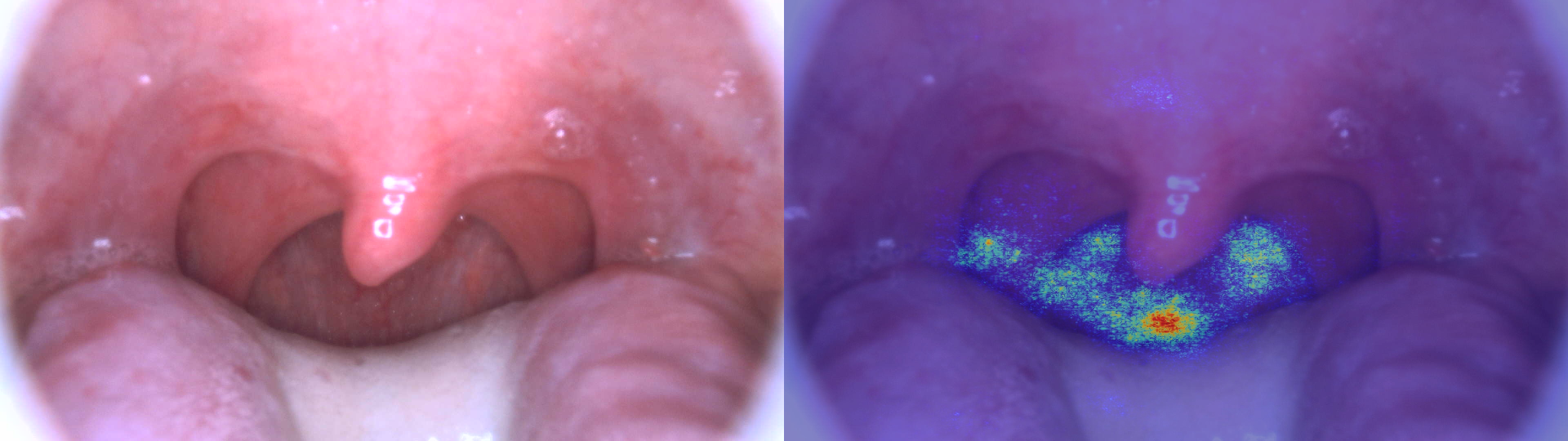


Tonsils


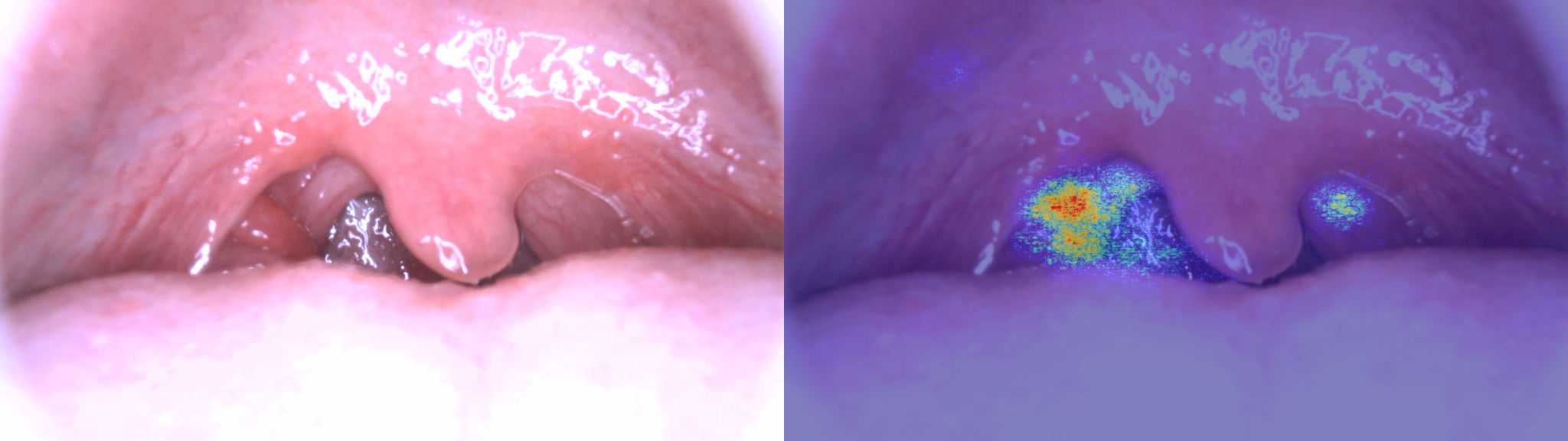


Palatal arch


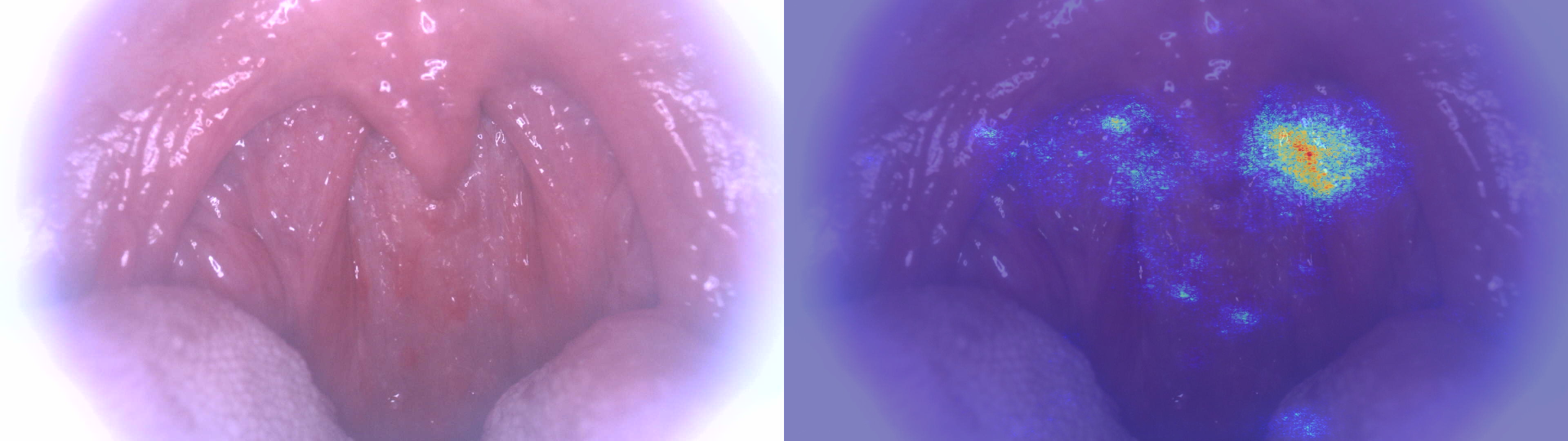


Negative case:


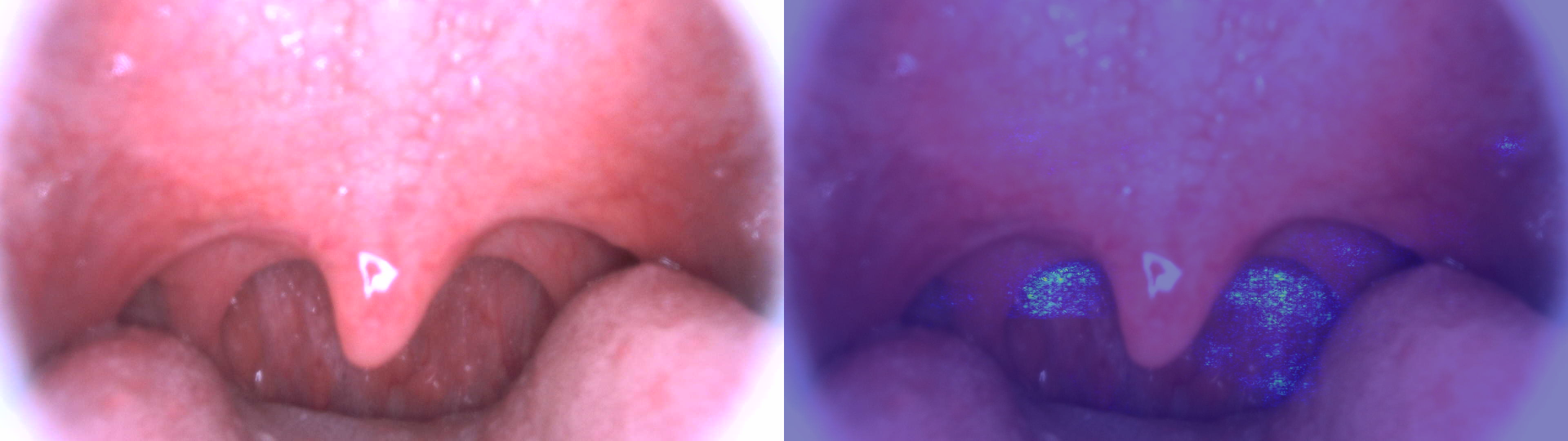


**Supplementary Figure 7. Proportion of patients with images highlighted by the AI model on each part of the pharynx in 100 RT-PCR-positive influenza cases and 100 RT-PCR-negative cases**

**
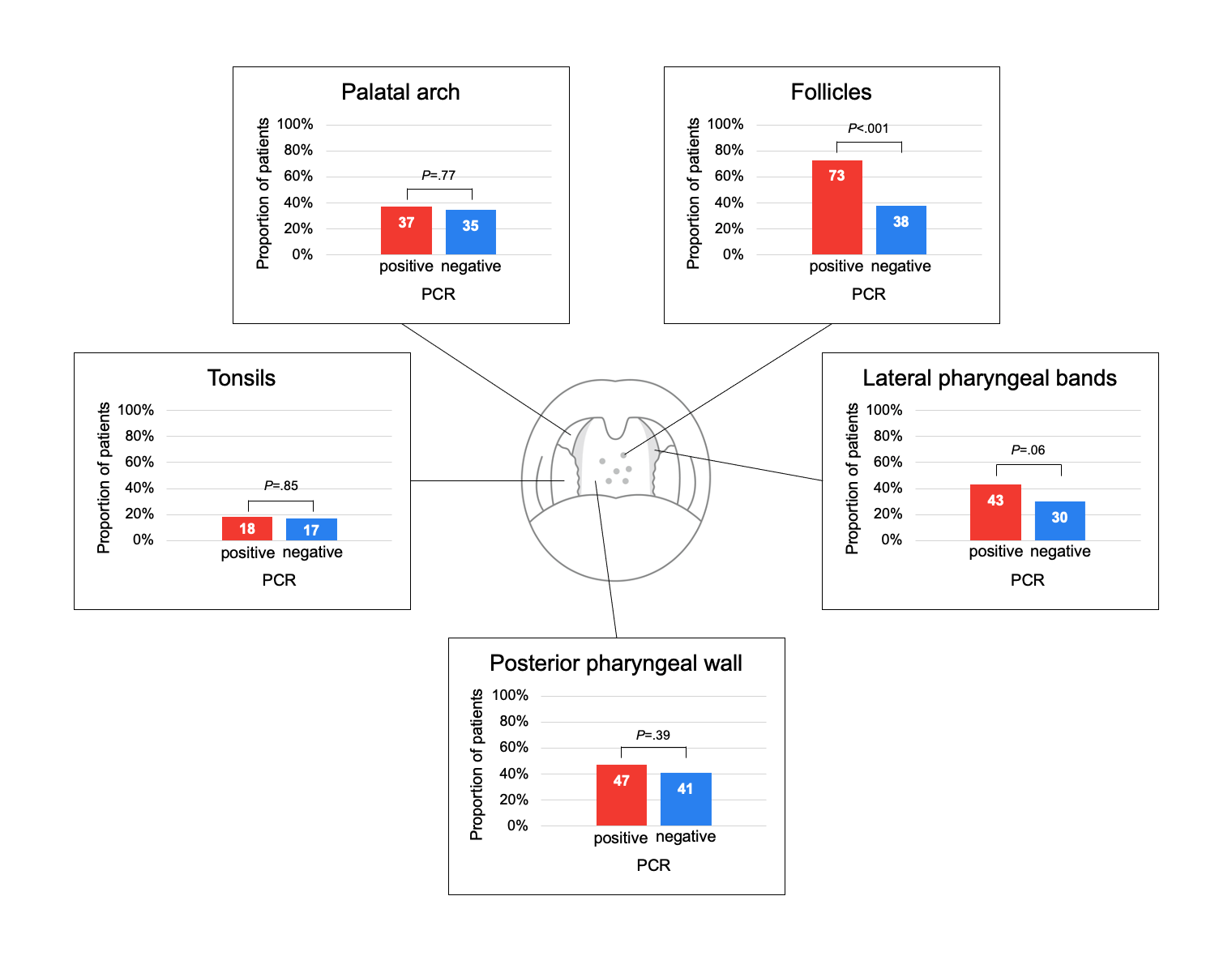
**

**Supplementary Table 2. Comparison of AUROC performance for the ensemble model and component models**

|  | Model name | AUROC |
| --- | --- | --- |
| Models using  pharyngeal images | MV-CNN | 0.74 |
| Models using  pharyngeal images  and clinical information | MM-CNN | 0.87 |
|  | CatBoost | 0.89 |
|  | LightGBM | 0.89 |
| Ensemble model | Ensemble | 0.89 |

Abbreviations: AUROC: area under the receiver operating characteristic curve, MV-CNN: multi-view convolutional neural network, MM-CNN: multi-modal convolutional neural network

**Supplementary Table 3. Comparison of the AUROC performance of the MV-CNN models with the proposed backbone model and that of various convolutional neural network backbones (five-fold cross-validation)**

|  | Backbone proposed in our final model | Other backbones | | | |
| --- | --- | --- | --- | --- | --- |
|  | SE-ResNeXt-50  (32x4d) | ResNet-50 | ResNeXt-50  (32x4d) | EfficientNet-B0 | DenseNet-121 |
| Fold 0 | 0.750 | 0.659 | 0.709 | 0.752 | 0.560 |
| Fold 1 | 0.747 | 0.674 | 0.722 | 0.747 | 0.542 |
| Fold 2 | 0.748 | 0.684 | 0.702 | 0.747 | 0.543 |
| Fold 3 | 0.748 | 0.614 | 0.723 | 0.734 | 0.558 |
| Fold 4 | 0.746 | 0.654 | 0.741 | 0.747 | 0.561 |
| Mean | 0.748 | 0.657 | 0.719 | 0.745 | 0.553 |

Abbreviations: AUROC: area under the receiver operating characteristic curve, MV-CNN: multi-view convolutional neural network

**Supplementary Table 4. The list of Aillis members who contributed to the study during the study period (Acknowledgement)**

Daichi Tanaka, Akiko Yamazaki, Takashi Yasumi, Yujiro Maeda, Akiho Sakuma, Miho Nakamura, Atsushi Fukuda, Masaki Nomura, Genji Kawano, Yukiko Oikawa, Kazuto Honda, Mizuki Watanabe, Yoshihiro Todoroki,

Keiichi Tamura, Ken Takahashi, Hiroshi Yoshihara, Quan Huu Cap, Kazuhiro Kawabata, Saho Kameyama, Chika Horike, Kouhei Akane, Takumi Shiokawa, Kazutaka Okuda, Yuji Ariyasu, Hirofumi Namba, Takashi Kinouchi, Teruyoshi Arikawa, Yu Oshima, Mari Kurashima, Kei Katsuno, Shimako Endo, Yu Nakayama
